# The National Strategies for and Challenges in Infection Prevention and Control of the Healthcare System in the Kingdom of Saudi Arabia (Review Study)

**DOI:** 10.3390/vaccines10081302

**Published:** 2022-08-11

**Authors:** Thamer Alslamah, Adil Abalkhail

**Affiliations:** Department of Public Health, College of Public Health and Health Informatics, Qassim University, Buraydah 52741, Saudi Arabia

**Keywords:** infection prevention and control, healthcare-associated infections, limited resources, healthcare workers, public health

## Abstract

Healthcare-associated infections (HAIs) lead to considerable morbidity. Prolonged hospital HAIs, also known as hospital infections or nosocomial infections, refer to infectious diseases that occur within 48 h of hospital admission, within three days of discharge or 30 days after having received healthcare. A total of 39 government-owned hospitals with a total of 10,822 beds providing the majority (59.9%) of Saudi Arabian healthcare were analyzed. Medicare and Medicaid use hospital data to track hospital performance on matters pertaining to IPC. In addition, many hospitals have limited capacity with which to develop the core components required to build an effective IPC programme. Hajj has been associated with the increased prevalence and spread of infectious diseases. These factors include changes in human demographics and behaviour, the effect of new technologies and industries, an increase in international travel and commerce, and breakdown resulting from public health measures. Overall public health service expenditure originates from the administration and services that are provided free of charge to all Saudi citizens.

## 1. Introduction

Healthcare-associated infections (HAIs) are a major issue for healthcare providers, infection control specialists, public health authorities, and patients. In developing countries, the risk of contracting HAIs is estimated to be up to 20 times higher [1], equivalent to an infection rate of up to 25% [2]. A recent Centre for Disease Control (CDC) report indicated that nursing homes report over 3 million HAIs per annum, some of which lead to patient death or disability [3]. HAIs are associated with an increase in mortality and morbidity, prolonged hospital stays, antibiotic resistance and increased healthcare costs [4]. It is estimated that HAIs result in between 44,000 and 98,000 unexpected deaths in the United States and the cost of dealing with these infections is estimated at USD 17–29 billion [5]. Data from national and multicentre studies show rates of HAIs in high-income countries ranging between 3.5% and 12% [6] and rates of between 5.7% and 19.1% in low-income countries.

## 2. Organisation of the Healthcare System in KSA

**Figure 1:**
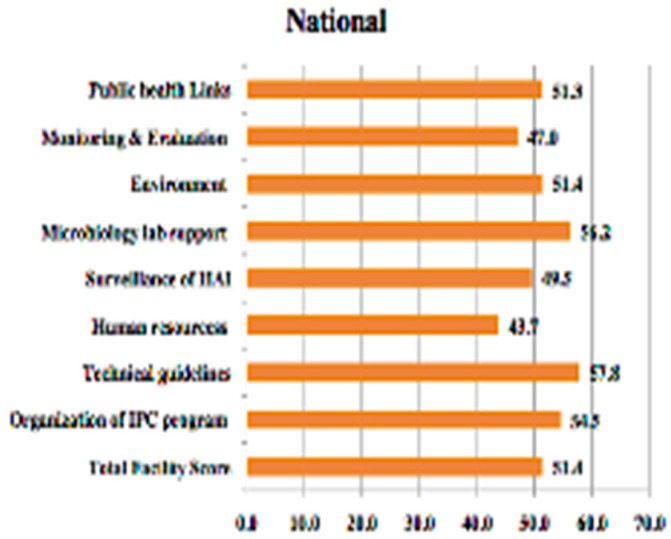
Total facility scores of IPC components at the national level [8].

KSA healthcare amenities have evolved over time. The first public health department was established in Mecca in 1925 [7]. The Ministry of Health of KSA was instituted in 1950. The Saudi healthcare system is composed of government-owned, public sector hospitals and privately-owned hospitals. The Ministry of Health is responsible for providing and financing government healthcare services. Hospitals and primary healthcare centres comprise 26% of Saudi Arabian health services. In addition to the Ministry of Health, other state bodies provide healthcare services to the general population, in addition to serving their employees and dependents. These bodies include the security forces (e.g., the National Guard health affairs, the security forces, and the army), the Royal Commission for Jubail and Yanbu health services, Johns Hopkins Aramco Healthcare, and school health units run by the Ministry of Education and the Red Crescent Society. State bodies cooperatively run 39 government-owned hospitals with a total of 10,822 beds providing the majority (59.9%) of Saudi Arabian healthcare (see Figure 1 below).

The country also has many private sector healthcare services. The private sector runs 125 hospitals in total with 11,833 beds and 2218 dispensaries and clinics that are primarily located in major towns and cities [9]. Overall, KSA currently boasts over 53,000 hospital beds per 1000 residents. When combined with the public sector, KSA’s bed per population has risen to over 70,000 beds [8]. The KSA healthcare system is divided into primary, secondary, and tertiary healthcare. Primary healthcare provides basic healthcare services to all people in KSA. Specialised treatment is offered at some private and public facilities, with referrals being made to hospitals such as the King Faisal Specialist Hospital, higher education hospitals (teaching hospitals) and research centres [9,10]. The public sector runs across all levels of healthcare, from primary healthcare to tertiary healthcare and high-risk and emergency services. Some government hospitals are specifically designed to complement each other instead of competing, with some hospitals being solely devoted to cancer and others to paediatric and maternity care, for example. Saudi Arabians are afforded free treatment as are government contractors, such as Aramco employees and their families [7,11]. The services offered by the public sector are incomparable with the general hospital healthcare services afforded by the private sector as public sector healthcare is given free at the point of delivery to all Saudi citizens.

The WHO has ranked the KSA health system as being the 25th best system in the world, ahead of most developed nations [12]. KSA’s healthcare workforce (HCW) is made up of 423,940 healthcare workers, of which 248,000 are physicians and nurses. However, a large percentage of physicians (73.4%) and nurses (49.7%) are foreigners from other Middle Eastern countries, Africa, and Asia, as Table 1 shows [7].

## 3. Infection Prevention and Control in Healthcare System in KSA

Infectious diseases have claimed many lives in KSA, although most infectious diseases can be treated or prevented. According to reports published by the MOH, brucellosis, chickenpox, and amoebic dysentery are the most chronic infections most easily transmitted among people in KSA [13]. There are IPC quality assurance departments that are established at a healthcare facility or institution with the task of implementing infection control programmes and guidelines. IPC is a new but growing discipline in KSA, and at the national level, the Saudi MOH is responsible for establishing several centres for disease control and prevention. For instance, the Command and Control Centre (CCC) was set up with the aims of enhancing the prevention of infections and establishing systems to track infections in KSA and worldwide. Agencies such as the Centres for Medicare and Medicaid use hospital data to track hospital performance on matters pertaining to IPC [9].

Every Saudi healthcare facility is also required to design, establish, and coordinate an IPC programme to identify, and reduce the risk of, infection acquisition and transmission amongst patients, staff, and visitors [14]. The MOH facilitated the establishment of infection control services in all its hospitals. Moreover, the MOH provides in-house training and field epidemiology training on infection control to all healthcare workers. The Saudi Council for Health Specialties also established a subspecialty training institution in infectious disease in internal medicine and paediatrics to fulfil extensive domestic demands. Therefore, there is now a large number of nationally trained Saudi internists and paediatricians [15]. Assiri et al. 2014 conducted a cross-sectional interview-based study to describe and assess the status of IPC programmes in KSA. The study focused on the status of the eight core components of IPC programmes that are deemed essential in strengthening capacity for the prevention of HAIs. The study calculated a combined score for the eight components for each healthcare facility. These eight core components include the organisation of IPC programmes, technical guidelines, human resources, surveillance of HAIs, microbiology lab support, environment, monitoring and evaluation, and public health links. The results indicated that the facilities’ combined scores ranged from 42% to 57%, as shown in Figure 1.

Examining the status of IPC programmes in different regions in KSA indicated that the combined scores for core components ranged from 42% to 67%, as shown in Table 2.

These results suggest that, in KSA, even though facilities have established infection control measures, their performance is suboptimal. IPC programmes have been shown to be clinically cost-effective [16]. Furthermore, they can provide significant cost savings through a reduction in HAIs, less time spent in hospital, and a reduction in microbial infection rates and treatments costs [17]. Based on the economic and human costs of HAIs, there is a need to implement relevant and sufficient resources for IPC initiatives.

## 4. Challenges in Hospital Infection Control in KSA

Based on some studies, KSA faces several challenges, limitations, and barriers to efficient infection control, all of which increase the number of health issues in the country leading to increased mortality and morbidity rates [16,18,19,20]. Most hospitals in KSA lack the required trained professionals to deal with infection control, and there is a need for awareness initiatives to deal with this deficiency. However, human resources, particularly those associated infectious diseases, are insufficient in KSA [16]. The infrastructure to aid infection control programmes in KSA is underdeveloped in comparison to global standards, as Rabaan et al. 2017 indicate. Many hospitals have limited capacity with which to develop the core components required to build an effective IPC programme. As such, there is a need to increase investment in resources to develop IPC components in accordance with acceptable standards. Such investments will reduce the costs of treating HAIs and decrease associated mortality and morbidity rates [16]. Accordingly, KSA healthcare facilities should evaluate the needs for infection control and establish an active infection control programme in order to reduce the various risks associated with HAIs and improve the safety of healthcare.

## 5. Hajj and Pilgrimages as a Challenge

KSA’s culture is defined by its Islamic heritage, its historical role as an ancient trade centre, and the Bedouin tradition. KSA’s society has evolved from traditional values, customs, hospitality, and styles of dressing to adapt to current forms of modernisation. However, traditional values are deeply rooted in Islamic teachings and Arab customs and are thus learned at an early age within families and in schools. The religious and cultural heritage of KSA poses particular challenges for the management of HAIs. The Hajj pilgrimage, which is an annual customary journey to the sacred location of Mecca, presents a major challenge to infection control in KSA. The Hajj event lasts for five days, involving around 3 million pilgrims and spanning several different locations. Hajj has been associated with the increased prevalence and spread of infectious diseases. Communicable diseases that have been found to increase in occurrence during the Hajj event include respiratory infections such as the MERS-CoV, influenza virus infections, meningococcal meningitis infection, tuberculosis, and blood-borne diseases including Hepatitis B, C and HIV [21,22]. Extended stays at each pilgrimage site, hot atmospheric temperatures, crowded accommodation, traffic jams and dust, and inadequate food preparation and storage all facilitate the transmission of airborne and waterborne infections.

The increased risk of contracting infectious diseases during Hajj has been associated with a wide range of factors, including extreme overcrowding, fatigue from travel and movement, high temperature and excessive humidity, dense dust air pollution, inadequate and contaminated nutrition, water scarcity, inadequate sewage management, and improper sanitary habits [22]. The congestion of people during Hajj has been found to increase carrier rates for Neisseria meningitidis to 80% of the population, which increases the risk of an epidemic outbreak [23]. For example, during the Hajj event in 1989, there was a large outbreak of meningitis in KSA. This prompted the Saudi government to demand that all attendees were vaccinated against meningitis before attending the annual Hajj event. Outbreaks of the serogroup W135 strain of meningococcal infection amongst 2000 and 2001 Hajj pilgrims and their families, both within the country and internationally, necessitated a change from a bivalent to quadrivalent vaccine [24]. The Ministry of Health reports a 57% increase in infectious disease related hospital admissions during the Hajj event every year, with pneumonia accounting for up to 39% of all hospital admissions during this time [23]. The influx of large numbers of temporary and permanent immigrants to KSA has contributed to continuingly high levels of tuberculosis (TB). The high percentage of elderly Hajj participants, and the high prevalence of TB in countries such as Singapore and Malaysia, where many of the pilgrims come from, have led to the resurgence of TB in the KSA [24]. The high volume and secondary transmission rates of these infections pose a challenge for the management of nosocomial infections. For instance, most of the infections that occur during and immediately after the Hajj event are preventable through proper vaccinations and protective hygiene measures. Seasonal influenza vaccination is recommended for people moving within the country, for individuals with pre-existing health conditions and for all healthcare staff working in healthcare facilities that serve Hajj participants [25]. Ensuring that all Hajj pilgrims are vaccinated against meningitis, pneumonia, and influenza remains a challenge for the Saudi government, as a result of the sheer number of participants and the remoteness of the country’s rural areas; moreover, if a pilgrim have any health problems during the Hajj event, one of the requirements for a pilgrim is a visa health insurance that covers any health problems during Hajj [2]. The financial and personnel resources necessary to increase the desired level of coverage, accessibility, and preparedness for free healthcare and vaccination services for the Hajj participants is enormous and a significant challenge for the Saudi government. 

The annual change in date for the Hajj festival further compounds the challenges faced by the government in preparing to handle infectious diseases common during the Hajj and other pilgrimages to religious sites in the country [26]. Challenges related to water scarcity for countries in the Gulf Peninsula continue to hamper efforts by the Saudi government towards instilling better hygiene practices for the Hajj season as well as improving public health standards, especially within urban areas [3].

## 6. The Increasing Burden of Emerging Infectious Disease

Over time, infectious diseases have spread widely across populations, regions, and across the world. Existing theories note that new infectious diseases will continue to be identified in the world [27]. Many infectious diseases arise from animal reservoirs and only affect human beings under certain circumstances. There are numerous factors that are associated with the emergence and spread of infectious diseases [28]. These factors include changes in human demographics and behaviour, the effect of new technologies and industries, an increase in international travel and commerce, and breakdown resulting from public health measures. Other factors include humans sharing the same environment as domestic and wild animals, as discussed by The Coronavirus Study Group of the International Committee on Taxonomy of Viruses (2013).

The emergence of new infectious diseases poses a challenge for healthcare facilities in controlling nosocomial infections. Sampathkumar 2014 describes a severe viral illness caused by a newly discovered coronavirus found in Middle Eastern countries, including KSA, and which was subsequently named the Middle East respiratory syndrome coronavirus (MERS-CoV). Based on the literature, 90% of MERS-CoV cases occurred in the Middle East region, with cases outside the region being reported only in people who had recently travelled to the Middle East, or had come into direct contact with someone who had recently travelled to the region [29]. The MERS-CoV illness has a high mortality rate as it is a communicable disease that can be easily transmitted between humans [28]. Fever and mild cough are among the first symptoms of the MERS-CoV illness. The breathing difficulties progress either rapidly or over several days based on the pre-medical conditions of infected patients resulting in dyspnoea and hypoxia, that is, inability to maintain oxygenation. The lungs, kidneys, and heart can be also infected by the virus, which leads to disruption in their functioning. The reverse transcriptase polymerase chain reaction (RT-PCR) test is used to diagnose the MERS-CoV illness. Using tests such as X-ray and CT-Scans may also be diagnostic [28]. Because of the lack of experimental data, there is no curative approach to treat the MERS-CoV infection other than offering support for disorders as, for example, oxygen supplementation and mechanical ventilation. The treatment should be adjusted based on the patients’ pre-medical conditions and there are different medications on trials.

However, there have not been conclusive benefits yet. As MERS-CoV does not spread rapidly, it is possible to take protective measures to prevent further outbreaks of the disease, such as avoiding close contact with infected people, wearing masks and gloves, washing hands regularly, and avoiding bodily fluid exchanges. Nevertheless, in hospitals and care centres, infected patients should be kept in airborne isolation rooms. Healthcare workers have to wear masks and gloves as well as cover their eyes when treating patients [28]. The MERS-CoV disease has become a major challenge for IPC as the foundation of the virus and the method of transmission have not yet been completely identified. However, studies show that the virus originates from single-humped camels in Egypt, Qatar, and KSA [28]. Such infections constitute a major barrier for prevention and control strategies in KSA.

## 7. Limited Budget Resources

The most difficult challenge faced by the Saudi Ministry of Health is the funding of healthcare services. Overall public health service expenditure originates from the administration and services that are provided free of charge to all Saudi citizens. This leads to substantial cost strains on the government, especially in the current context of population growth, the high cost of technology, and increasing awareness of health and disease [7]. According to the WHO, KSA allocates 22.75% of total GDP to the healthcare sector [28], although resources are unevenly distributed with a substantial percentage (51.34%) of spending attributed to bigger communities [28]. Although born out of the desert two generations ago, KSA has made tremendous progress in both its private and public healthcare system. Fully aware of the exigencies of establishing a strong healthcare system, budget allocations to healthcare have consistently increased.

Despite continued efforts to become globally competitive and meet the highest of standards, this goal has not yet been achieved. Nevertheless, KSA has become a recognised healthcare leader within the Arab Middle East region. The Saudi Ministry of Health continues to evolve and develop in its aim to meet the highest global healthcare quality levels [30]. According to a report published by the MOH, over the period from 2015 to 2016, the Ministry of Finance allocated SAR 59,985.36019 for healthcare spending, rising to SAR 62,342.539 in 2016 [28]. However, as the Kingdom of Saudi Arabia Budget Report [31] notes, neither of the figures were enough to fulfil the Ministry’s plan to make KSA the regional centre for healthcare. Therefore, starting in 2017, the Ministry of Finance started allocating 19% of the national budget to healthcare and education. This translated into USD 26.3 billion for 2017 and, as it was still considered insufficient, the 2018 budget was increased to USD 34.3 billion [31]. Saudi Arabians receive free treatment in all public hospitals, clinics, and outpatient centres, but foreigners cannot enter the kingdom for work purposes or for any length of time, if they do not have health coverage with a major insurer [7].

## 8. Conclusions

Saudi healthcare facility is required to design, establish, and coordinate an IPC programme to identify, and reduce the risk of, infection acquisition and transmission amongst patients, staff, and visitors. Healthcare facilities should evaluate the needs for infection control and establish an active infection control programme in order to reduce the various risks associated with HAIs and improve the safety of healthcare. Infections constitute a major barrier for prevention and control strategies in KSA.

## Figures and Tables

**Table 1:** Healthcare professionals employed in the KSA as of 2017 [8].

Categories of Healthcare Professionals	Total Workforce	Percentage of Saudi Workforce	Percentage of Foreign Workforce
**Allied Health**	98,074	29.5	71.5
**Personnel**			
**Pharmacists**	185,693	36.7	63.3
**Nurses**	28,312	22.2	77.8
**Physicians**	111,861	74.7	25.3
**Total**	**423,940**	**44.1**	**56.9**

**Table 2:** Illustration of key IPC components and their values in different parts of the KSA [8].

Infection Control and Prevention Components	North Area	East Area	Central Area	West Area	South Area	National Level
**Organisation of IPC Program**	50.6%	50.1%	61.7%	57.1%	55.6%	54.5%
**Technical Guidelines**	53.6%	53.1%	64.9%	60.8%	57.3%	57.8%
**Human Resources**	39.6%	42.1%	46.7%	46.0%	45.0%	43.7%
**Surveillance of HAI**	43.9%	44.6%	55.5%	52.8%	52.3%	49.5%
**Microbiology Lab Support**	49.3%	52.8%	63.7%	60.5%	57.8%	56.2%
**Environment**	45.8%	42.0%	59.8	57.4%	53.4%	51.4%
**Monitoring and Evaluation**	40.0%	43.3%	54.4%	50.0%	50.6%	47.0%
**Public Health Links**	44.3%	42.6%	59.0%	54.1%	57.8%	51.3%

## Data Availability

Not applicable.
